# Comparison of cisplatin-based versus standard preoperative chemotherapy in patients with operable triple-negative breast cancer: propensity score matching and inverse probability of treatment weighting analysis

**DOI:** 10.1007/s10549-023-07163-z

**Published:** 2023-12-21

**Authors:** Ayane Yamaguchi, Kosuke Kawaguchi, Kana Kawanishi, Yurina Maeshima, Akiyoshi Nakakura, Tatsuki R. Kataoka, Sachiko Takahara, Shunsaku Nakagawa, Atsushi Yonezawa, Masahiro Takada, Masahiro Kawashima, Nobuko Kawaguchi-Sakita, Takeshi Kotake, Eiji Suzuki, Hanako Shimizu, Masae Torii, Satoshi Morita, Hiroshi Ishiguro, Masakazu Toi

**Affiliations:** 1 Department of Breast Surgery, Kyoto University Graduate School of Medicine, 54 Shogoin-Kawaharacho, Sakyo-Ku, Kyoto, 606-8507 Japan; 2 Department of Breast Surgery, Kobe City Nishi-Kobe Medical Center, 5-7-1, Kojidai, Nishi-Ku, Kobe, 651-2273 Japan; 3 Department of Biomedical Statistics and Bioinformatics, Kyoto University Graduate School of Medicine, 54 Shogoin-Kawaharacho, Sakyo-Ku, Kyoto, 606-8507 Japan; 4 Department of Molecular Diagnostic Pathology, Iwate Medical University, 1-1-1 Idaidori, Yahaba-Cho, Shiwa-Gun, Iwate 028-3694 Japan; 5 Department of Breast Surgery, Tazuke Kofukai, Medical Research Institute, Kitano Hospital, 2-4-20 Ohgimachi, Kita-Ku, Osaka, 530-8480 Japan; 6 Department of Clinical Pharmacology and Therapeutics, Kyoto University Hospital, 54 Shogoin-Kawaharacho, Sakyo-Ku, Kyoto, 606-8507 Japan; 7 Department of Therapeutic Oncology, Kyoto University Graduate School of Medicine, 54 Shogoin-Kawaharacho, Sakyo-Ku, Kyoto, 606-8507 Japan; 8 Department of Breast Surgery, Kobe City Medical Center General Hospital, 2-1-1 Minatojimaminami-Cho, Chuo-Ku, Kobe, 650-0047 Japan; 9 Department of Breast Surgery, Japanese Red Cross Wakayama Medical Center, 4-20 Komatsubara-Dori, Wakayama City, 640-8558 Japan; 10 Breast Oncology Service, Saitama Medical University International Medical Center, 1397‐1 Yamane, Hidaka, Saitama, 350‐1298 Japan; 11 Tokyo Metropolitan Cancer and Infectious Disease Center, Komagome Hospital, 3-18-22, Honkomagome, Bunkyo-Ku, Tokyo, 113-8677 Japan

**Keywords:** Cancer, Neoadjuvant chemotherapy, Platinum, Residual cancer burden

## Abstract

**Purpose:**

The efficacy of carboplatin is non-equivalent to that of cisplatin (CDDP) for various tumor types in curative settings. However, the role of CDDP in operable triple-negative breast cancer (TNBC) patients remains unknown. We conducted a multicenter observational study to examine the effects of CDDP added to preoperative chemotherapy in patients with TNBC.

**Methods:**

This retrospective study consecutively included previously untreated patients with stage I–III TNBC treated with preoperative chemotherapy with or without CDDP. The primary endpoint was distant disease-free survival (DDFS). Propensity score matching (PSM) and inverse probability of treatment weighting (IPTW) were used to minimize confounding biases in comparisons between the two groups.

**Results:**

A total of 138 patients were enrolled in the study. Of these, 52 were in the CDDP group and 86 in the non-CDDP group. DDFS was significantly better in the CDDP group than in the non-CDDP group (unadjusted hazard ratio (HR) 0.127 and *p* < 0.001, PSM HR 0.141 and *p* < 0.003, IPTW HR 0.123 and* p* =  < 0.001). Furthermore, among the patients with residual cancer burden (RCB) class II/III, DDFS was better in the CDDP group than in the non-CDDP group (unadjusted HR 0.192 and *p* = 0.013, PSM HR 0.237 and* p* = 0.051, IPTW HR 0.124 and *p* = 0.059).

**Conclusion:**

Our study showed that CDDP-containing regimens achieved favorable prognoses in patients with operable TNBC, especially for the RCB class II/III population. Confirmative studies are warranted to elucidate the role of CDDP in TNBC treatment.

**Supplementary Information:**

The online version contains supplementary material available at 10.1007/s10549-023-07163-z.

## Introduction

Triple-negative breast cancer (TNBC) carries a greater risk of distant recurrence and mortality than other breast cancer subtypes [1, 2]. Notably, most TNBCs are of the basal subtype based on the PAM50 gene expression profile, which has been linked to worse recurrence-free survival and overall survival (OS) in patients with recurrent disease (RD) following neoadjuvant chemotherapy (NAC) [1, 3]. The intrinsic genomic instability observed in specific TNBC cells, particularly those of the basal subtype, is a consequence of inadequate DNA repair systems, which may increase the sensitivity to platinum-based chemotherapy agents [4, 5].

Platinum agents (carboplatin (CBDCA) and cisplatin(CDDP)) are cytotoxic DNA-damaging compounds that cause DNA strand breaks and possible cell apoptosis; this unique mechanism of action renders these agents particularly active against cancer cells with DNA repair deficiency, such as those harboring deleterious mutations in the *BRCA* genes [6]. Given the molecular mechanism contributing to the increased vulnerability of TNBC cells to DNA-damaging compounds [7], several clinical studies have examined the potential role of platinum drugs as a therapeutic option for TNBC patients.

Adding immune checkpoint inhibitors affects the preoperative and postoperative treatment of TNBC considerably [8]. Combining pembrolizumab and anticancer agents has become the standard of care in preoperative cStage II/III TNBC treatment. However, controversy exists around whether adding carboplatin-based chemotherapy should be a standard treatment for stages II and III TNBC. Recent clinical studies have shown that adding platinum-based chemotherapy to neoadjuvant regimens may improve the likelihood of a complete pathological response (pCR) [9, 10]. However, a recent phase 3 randomized study showed that platinum agents (carboplatin for 88% of patients) did not improve outcomes in patients with a basal subtype TNBC residual tumor after NAC and were associated with more severe toxicity than capecitabine [11].

CBDCA possesses a bidentate dicarboxylate ligand instead of two chloride ligands found in CDDP as the leaving groups. CBDCA is less reactive and has slower DNA-binding kinetics than CDDP, but both compounds form the same reaction products in vitro at equal dosages. However, unlike CDDP, CBDCA may be vulnerable to other pathways [12]. The differences in the chemical structures of CDDP and CBDCA and their pharmacokinetics in intravenous infusion are shown in Table S1 of Online Resource 1[13]. Although CBDCA forms the same reaction products in vitro as CDDP at doses, it shows lower reactivity and slower DNA-binding kinetics. This diminished reactivity limits the formation of protein–CBDCA complexes, which are excreted. Accordingly, CBDCA is considered to be less potent than CDDP, with a 1/8 to 1/45 in potency depending on the type of cancer. Therefore, the standard clinical dosage of CBDCA is usually determined at a 4:1 ratio compared to CDDP [12]. CDDP has been incorporated into the standard anticancer drug regimen for curative intent in various tumor types, such as lung, gastrointestinal, genitourinary, and other cancers. CBDCA has been substituted with CDDP due to its ease of administration and reduced toxicity in certain situations, especially in palliative settings, such as for treating metastatic non-small cell lung cancer [14]. CDDP and CBDCA are unavailable under Japanese medical insurance. We have been using CDDP-based NAC for TNBC rather than CBDCA-based since we assume CBDCA is non-equivalent to CDDP based on clinical evidence for head and neck, esophageal, and germ cell tumors [15]. With appropriate supportive measures, the administration of CDDP and its toxicity have become manageable recently. Therefore, we hypothesized CDDP shows better therapeutic efficacy than standard therapy, including CBDCA. We decided to include a small number of patients who used CBDCA in the non-CDDP group because we also focused on the difference between CDDP and CBDCA.

Based on the limitations of currently available evidence on the degree of benefits and risks of adding platinum compounds to NAC for TNBC, this retrospective study was conducted to examine the difference in outcomes between patients who received NAC with or without CDDP.

## Material and methods

### Study proportion

This retrospective study consecutively included previously untreated patients with stage I–III TNBC treated with preoperative chemotherapy, including CDDP, between 2007 and 2019 at Kyoto University Hospital. As CDDP is considered off-label use for preoperative chemotherapy for TNBC in Japan, we received approval from the Ethics Committee of Kyoto University Graduate School and Faculty of Medicine for this study between 2007 and 2016. From 2016 onward, we followed the usage criteria of the drug department of Kyoto University Hospital since Ethics Committee approval was no longer required for the use of the CDDP regimen. Following consultation with our statistical expert, we concluded that the sample size of the control group should ideally be approximately twice that of the CDDP group. To ensure generalizability, the control group consisted of patients with TNBC who received regimens that did not include CDDP at Kyoto University Hospital, the Japanese Red Cross (JRC) Wakayama Medical Center, and Tazuke Kofukai Medical Research Institute Kitano Hospital. We consecutively included patients treated between 2007 and 2019 at Kyoto University Hospital, including the CDDP group, and patients treated between 2011 and 2019 at JRC Wakayama Medical Center and Kitano Hospital.

All patients of Kyoto University Hospital who met the following criteria were enrolled in the CDDP group: considered tolerant of CDDP treatment, consented to the use of CDDP, and received at least two doses of CDDP.

The inclusion criteria were as follows: female sex; confirmed diagnosis of TNBC, defined as < 10% positivity for both estrogen receptor and progesterone receptor by routine immunohistochemistry (IHC) [16] and human epidermal growth factor receptor 2 (HER2) IHC score of 0 and 1, or lack of HER2 amplification determined by fluorescence in situ hybridization or dual-color in situ hybridization (HER2/CEP17 ratio < 2.0); clinical stage (cStage) I–III; not under treatment for other cancers; undergoing surgery after preoperative chemotherapy; and receiving at least two doses of anticancer drugs throughout the regimen. Interrupted treatment due to allergic reactions did not count as a single visit. All patients who met the following criteria were enrolled in the CDDP group: considered tolerant of CDDP treatment, consented to the use of CDDP, and received at least two doses of CDDP. In the CDDP group, patients must have received a regimen containing CDDP for at least two cycles. Any patient that received only a single dose of CDDP was excluded from all analyses. The patients that received a regimen containing CBDCA were included in the non-CDDP group.

Patients were excluded from all analyses if they met the following conditions: male, did not undergo surgery after chemotherapy, received only one dose of any chemotherapy, had stage IV breast cancer, or were being treated for other cancers.

### Clinicopathological data

Clinicopathological data such as clinical and pathological stage, nuclear grade, histological grade, axillary lymph node involvement, Ki-67 proliferation index, preoperative chemotherapy regimen, postoperative treatment, and Grade 3 or above severe toxicities or those related to chemotherapy that required dose reduction, including a reduction in the variety of anticancer drugs used or discontinuation of chemotherapy, which results in regimen interruption or change, were obtained from the electronic medical records. Toxicity was graded according to the National Cancer Institute Common Toxicity Criteria version 5.0.

### Evaluation of pathological response

A pCR was defined as the absence of residual invasive cancer in the breast or lymph nodes. According to Symmans et al., the extent of RD in surgical specimens after preoperative chemotherapy was classified into four residual cancer burden (RCB) classes based on the RCB index: pCR with no residual invasive and non-invasive tumors both in the breast and lymph nodes (RCB-0), minimal RD (RCB-I), moderate RD (RCB-II), or extensive RD (RCB-III) [17].

### Evaluation of tumor-infiltrating lymphocytes (TILs)

TILs were evaluated in patients for whom samples were available on one representative hematoxylin–eosin-stained section of a biopsy specimen before NAC, according to the recommendations of the International Immuno-Oncology Biomarker Working Group [18].

### Primary and secondary endpoints

The primary endpoint was distant disease-free survival (DDFS) and the secondary endpoints were event-free survival (EFS), overall survival (OS), and liver metastasis-free survival (LMFS). DDFS was defined as the time to the occurrence of distant metastasis. EFS was defined as the time to the occurrence of the first of the following events: local, regional, or distant recurrence following surgery or death from any cause. Occurrence of a second primary breast cancer or any other non-breast primary cancer was not included in the EFS events. OS was defined as the time to death. LMFS was defined as the time to occurrence of liver metastasis. Patients lost to follow-up or those without critical events at their most recent follow-up were excluded. All survival outcomes were measured from the date of the initiation of preoperative chemotherapy to the date of the first event.

Two sensitivity analyses were conducted. First, eligible patients were excluded if they took oral fluorouracil (5-FU; capecitabine, S-1, or UFT) postoperatively, regardless of the time. Second, EFS was redefined as the time to the first occurrence of the following events: local, regional, or distant invasive or non-invasive recurrence of breast cancer following surgery; a new breast cancer or secondary malignancy; or death from any cause. Sensitivity analysis was performed further to examine the impact of CDDP on patient prognosis. Sensitivity analysis 1 was performed to exclude the potential impact of postoperative oral 5-FU therapy, which is known to improve the prognosis of patients with TNBC [19, 20]. Sensitivity analysis 2 was performed to exclude primary TNBC recurrence and to confirm that the inclusion of 2nd primary breast cancer or cancer of other organs in the event did not change the tendency of the analysis.

### Measurement of serum platinum concentrations in patients receiving regimens containing CDDP or CBDCA

Among the patients who provided comprehensive consent for inclusion in the study “Exploratory Study of Molecular Biological Mechanisms Involved in Breast Cancer Microenvironment Formation Using Biological Samples” conducted at Kyoto University Hospital, all patients who had received CDDP- or CBDCA-based treatment regimen and for whom serum was available were included for analysis of serum platinum concentration, regardless of the subtype of breast cancer. The details of these patients are provided in Table S2 of Online Resource 1. All 15 patients who received a regimen including CDDP had TNBC, whereas nine of the 10 patients who received the CBDCA regimen had HER2-positive breast cancer and the remaining patient was being treated for recurrent TNBC. Platinum concentrations in the serum of these 25 patients were determined using a mass spectrometer (ICP-MS Agilent 7700). For both the CDDP and CBDCA groups, the elimination rate constant was calculated from the correlation between the time since the last dose and the platinum concentration in the serum, and the half-life period was subsequently calculated. A semi-logarithmic graph was plotted with time since the last dose on the horizontal axis and blood concentration on the vertical axis, and the elimination rate constant was derived from multiplying the gradient of the graph by a negative value and the half-life period was calculated by ln(2)/elimination rate constant.

### Statistical analysis

The variables used for matching in the statistical analysis were the minimum necessary and included only those considered to have a clear impact on prognosis. Propensity score (PS) matching was used to minimize confounding biases in comparisons between the CDDP and non-CDDP groups. For all patients, the potential confounding factors for estimating PS were specified as follows: age, cT (≥ 2 or ≤ 1), and cN (positive or negative). For patients classified as RCB-II or RCB-III, potential confounding factors for estimating the PS were specified as follows: age, cT (≥ 2 or ≤ 1), cN (positive or negative), and RCB-II or III. The patients were matched using the nearest-neighbor method with a 0.2 increment. Analyses were also conducted using the estimated PS with inverse probability of treatment weighting (IPTW). For IPTW analysis, the standard errors were estimated using a robust sandwich variance estimator. The balance of all confounding factors was assessed using standardized differences. Because this was an exploratory study, the sample size was the total number of cases at the participating sites during the same period.

The Kaplan–Meier method was used to estimate DDFS, EFS, and OS, and the log-rank test was used to compare survival curves between groups. Statistical analyses were performed using JMP (version 16.2.0, SAS Institute, Inc., Cary, NC, USA), GraphPad Prism (version 6.07, GraphPad Software, Inc., San Diego, CA, USA), and SAS (version 9.4, SAS Institute, Inc., Cary, NC, USA) and statistical significance was set at *p* < 0.05.

## Results

### Patient characteristics

**Fig. 1:**
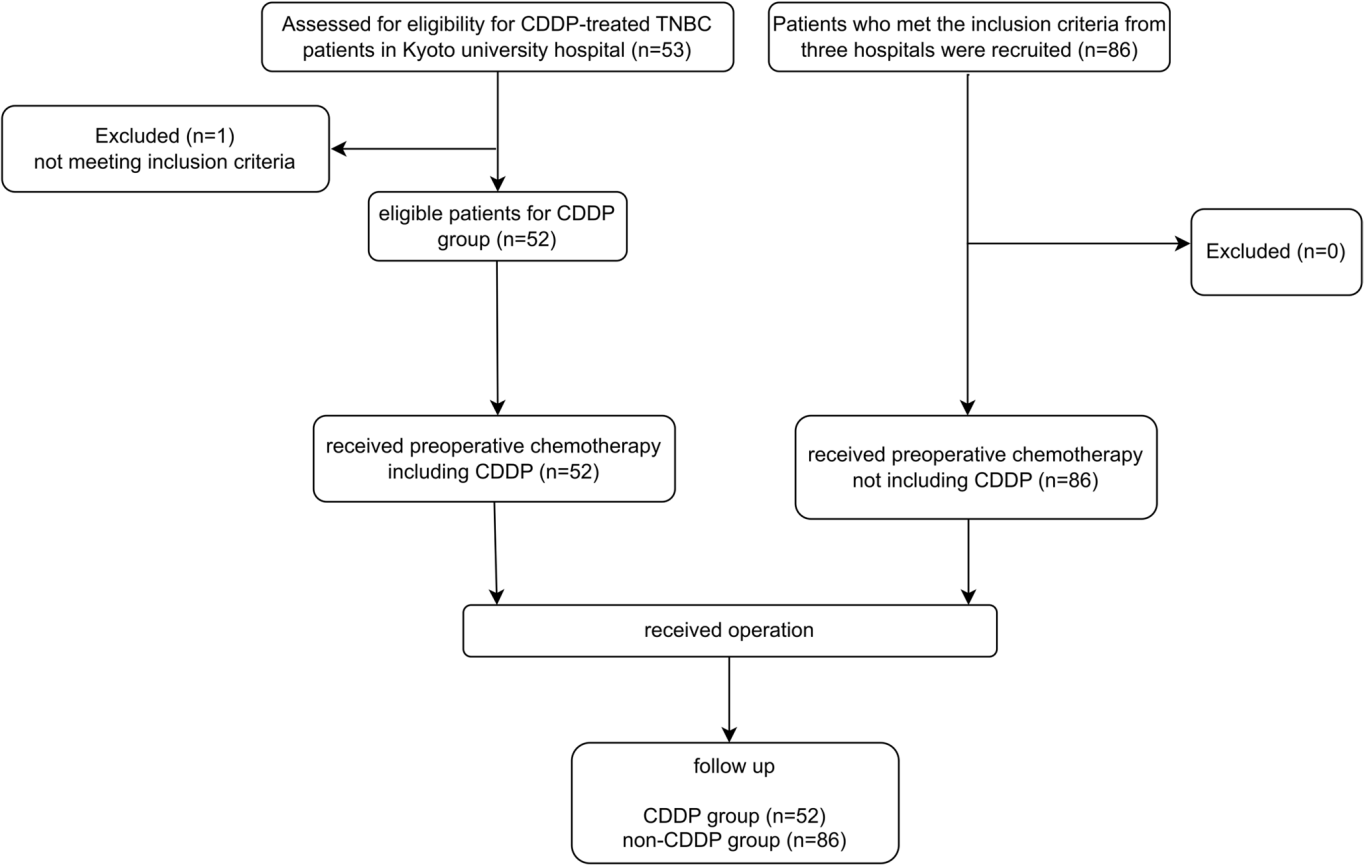
Flowchart of the patient recruitment process. Patients were recruited from three hospitals, with 52 in the cisplatin (CDDP) group and 86 in the non-CDDP group. The number of cases was considered necessary and sufficient as a sample size

Figure 1 shows a flowchart of the patient recruitment process. Fifty-three TNBC patients were treated with CDDP, 52 of whom met inclusion criteria, except one patient who received only a single dose of a regimen containing CDDP. As a control group, 86 patients who received preoperative chemotherapy with a regimen that did not include CDDP from Kyoto University Hospital, JRC Wakayama Medical Center, and Kitano Hospital were recruited. All patients received the operation and were followed up postoperatively. The median follow-up was 5.0 years.

The patient characteristics are summarized in Table 1. There were no significant differences between the CDDP and non-CDDP groups except for age, with significantly older patients in the non-CDDP group. Notably, more patients with cT4 were in the non-CDDP group; however, more patients with cN3 were in the CDDP group, resulting in no overall difference in the percentage of patients with cStage III disease between the two groups. Between groups, there was no difference in the completion rates of chemotherapy regimens and postoperative radiation or oral 5-FU therapy, including capecitabine, S-1, and UFT.

**Table 1:** Clinical characteristics of patients in the two groups

Characteristics	CDDP (*n* = 52)		Non-CDDP (*n* = 86)		P value
	*n*	%	*n*	%	
Age					
≤ 50	30	57.7	32	37.2	0.022
> 50	22	42.3	54	62.8	
range (median)	29–73 (48.5)		31–76 (55.5)		
cT stage					
1c	16	30.8	25	29.1	0.231
2	30	57.7	44	51.2	
3	3 5	9.6	7	8.1	
4b–d	1	1.9	10	11.6	
cN stage					
0	32	61.5	57	66.3	0.334
1	13	25	25	29.1	
2	2	3.8	2	2.3	
3a-b	4	7.7	2	2.3	
cStage					
I	11	21.2	21	24.4	0.941
IIA–B	31	59.6	49	57	
IIIA–C	10	19.2	16	18.6	
ER status					
0	38	73.1	73	84.9	0.121
1–9%	14	26.9	13	15.1	
*BRCA/* HRD status					
*BRCA1/2 mutation* ( +) or HRD positive	8	15.4	3	3.5	0.713
No mutation and HRD negative	17		32.7	11	
Unknown	27	51.9	72	83.7	
Chemotherapy regimen					
Anthracycline-based	45	86.5	72	83.7	0.808
Taxane-based	50	96.2	78	90.7	0.319
Complete chemotherapy	41	78.8	72	83.7	0.5
Radiotherapy					
Yes	35	67.3	64	74.4	0.436
None	17	32.7	22	25.6	
Adjuvant oral 5-FU					
Yes	8	15.4	11	12.8	0.619
No	42	80.8	73	84.9	
Unknown	2	3.8	2	2.3	

Oral 5-FU: capecitabine, S-1, and UFT, ER:estrogen receptor, HRD:homologous recombination deficiency

The regimen for the CDDP group was based on the following: CDDP (75 mg/m^2^) in combination with docetaxel (75 mg/m^2^) (TP) regimen every 3 weeks for four cycles, regarding regimens for non-small cell lung cancer [21], followed by the anthracycline regimen every 3 weeks for four cycles. Forty patients (76.9%) completed the treatment regimen as prescribed. Seven patients (13.2%) received three or fewer cycles of the TP regimen due to side effects or other reasons. One patient (1.8%) underwent six cycles of the TP regimen because of the good efficacy of the treatment after four TP cycles and the patient’s preference to continue the same regimen. Three patients (5.7%) used Gemcitabine (1000–1250 mg/m^2^) instead of docetaxel because of an allergic reaction. Gemcitabine was administered on days 1, 8, and 15, with 4 weeks as one cycle with reference to a regimen for non-small cell lung cancer [21]. Five patients (8.6%) received a combination of CDDP (50 mg/m^2^) and doxorubicin (45–50 mg/m^2^) with or without cyclophosphamide (500 mg/m^2^), with reference to regimens for endometrial carcinoma [22].

As shown in Table 1, the usage of anthracyclines and taxanes in the CDDP group was not different from that of the non-CDDP group. Toxicity profiles of the CDDP groups above Grade 3 and toxicity related to dose intensity are shown in Table S3 of Online Resource 1. Grade 3 or higher overall adverse events were more common in the non-CDDP group compared to the CDDP group (13.2% vs.7.9%). In terms of hematologic toxicity, anemia occurred in 2% of patients in the CDDP group, compared to 0.5% in the non-CDDP group. On the other hand, neutropenia was more common in the non-CDDP group (8.2% vs. 2.2%). Among non-hematologic toxicities, nausea and vomiting were more common in the non-CDDP group (5.2% vs. 0.7%). Adverse events leading to dose reduction were also more common in the non-CDDP group (7.0% vs. 1.2%), while the incidence of adverse events leading to discontinuation was slightly higher in the CDDP group (2.7% vs. 1.0%).

The RCB class and pCR ratio for the CDDP and non-CDDP groups are shown in Fig S1 of Online Resource 1. The CDDP group had significantly more RCB-0 and fewer RCB-II groups than the non-CDDP group. The pCR rate in the CDDP group was 55.8% and 31.4% in the non-CDDP group.

The characteristics of patients with distant metastatic recurrence are shown in Table S4 of Online Resource 1. In the CDDP group, no distant metastatic recurrence was observed in the patients with clinical stage I or II diseases. Among 25 patients in the non-CDDP group with distant metastatic recurrence, 13 were at cStage I or II.

### Survival comparison between the CDDP and non-CDDP groups

**Fig. 2:**
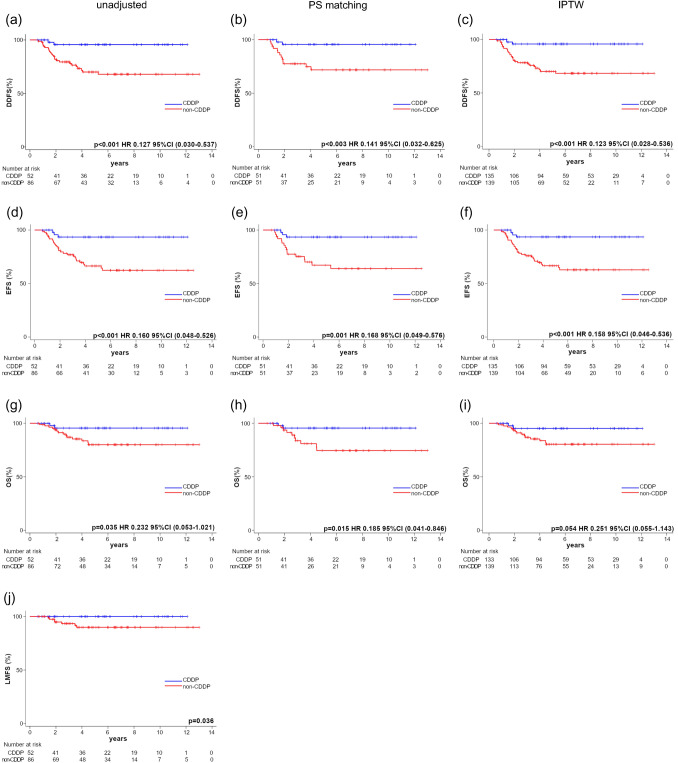
Survival curves of the CDDP and non-CDDP groups for **a**–**c** distant disease-free survival (DDFS), **d**–**f** event-free survival (EFS), **g**–**i** overall survival (OS), and **j** liver metastasis-free survival (LMFS). **a**, **d**, **g**, and **j** Show unadjusted analyses, **b**, **e**, and **h** are adjusted by the propensity score matching method, and (**c**, **f**, and **i**) are adjusted by the inverse probability of the treatment weighting method. Each survival curve was compared using the log-rank method

The survival curves for DDFS, EFS, and OS of the CDDP and non-CDDP groups are presented in Fig. 2. Table 2 shows the patient characteristics after adjustment for PS matching and IPTW.

**Table 2:** Patients’ characteristics with and without adjustment for propensity score (PS) matching and inverse probability of treatment weighting (IPTW) used in survival analyses

Characteristics	Overall			PS matching			IPTW		
	CDDP (*n* = 52)	Non-CDDP (*n* = 86)	Standardized difference	CDDP (n = 51)	Non-CDDP (*n* = 51)	Standardized difference	CDDP (*n* = 135)	Non-CDDP (*n* = 139)	Standardized difference
Age									
Mean (SD)	48.5 (10.58)	55.0 (11.58)	0.59	48.5 (10.68)	48.8 (9.90)	0.03	51.7 (17.05)	52.3 (15.42)	0.04
cT									
2–4	36 (69.2%)	61 (70.9%	0.04	36 (70.6%)	34 (66.7%)	0.08	96 (71.4%)	98 (70.3%)	0.02
1	16 (30.8%)	25 (29.1%))	0.04	15 (29.4%)	17 (33.3%)	0.08	39 (28.6%)	41 (29.7%)	0.02
cN									
Positive	20 (38.5%)	29 (33.7%)	0.1	19 (37.3%)	17 (33.3%)	0.08	51 (37.6%)	51 (36.3%)	0.03
Negative	32 (61.5%)	57 (66.3%)	0.1	32 (62.7%)	34 (66.7%)	0.08	84 (62.4%)	89 (63.7%)	0.03

Regarding DDFS and EFS, the log-rank test showed a significantly better prognosis for the CDDP group than the non-CDDP group in the unadjusted, PS matching, and IPTW analyses. In the unadjusted analysis, the 3-year and 5-year DDFS rates were 95.6% for both the CDDP group and 78.9% and 69.4%, respectively, in the non-CDDP group. In the unadjusted analysis, the 3-year and 5-year EFS rates were 93.5% in the CDDP group and 77.0% and 66.4%, respectively, in the non-CDDP group. For OS, the log-rank test also showed a significantly better prognosis for the CDDP group than for the non-CDDP group in the unadjusted and PS matching analyses. Contrastingly, the IPTW analysis showed a similar trend but did not reach statistical significance. The 3-year and 5-year OS rates in the unadjusted analysis were 95.6% in the CDDP group and 86.9% and 80.0%, respectively, in the non-CDDP group. Regarding LMFS, the log-rank test showed a significantly better prognosis in the CDDP group than in the non-CDDP group. The HR could not be calculated for LMFS, because there was no liver metastatic recurrence in the CDDP group.

### Survival comparison of CDDP and non-CDDP groups for RCB-II/III patients

**Fig. 3:**
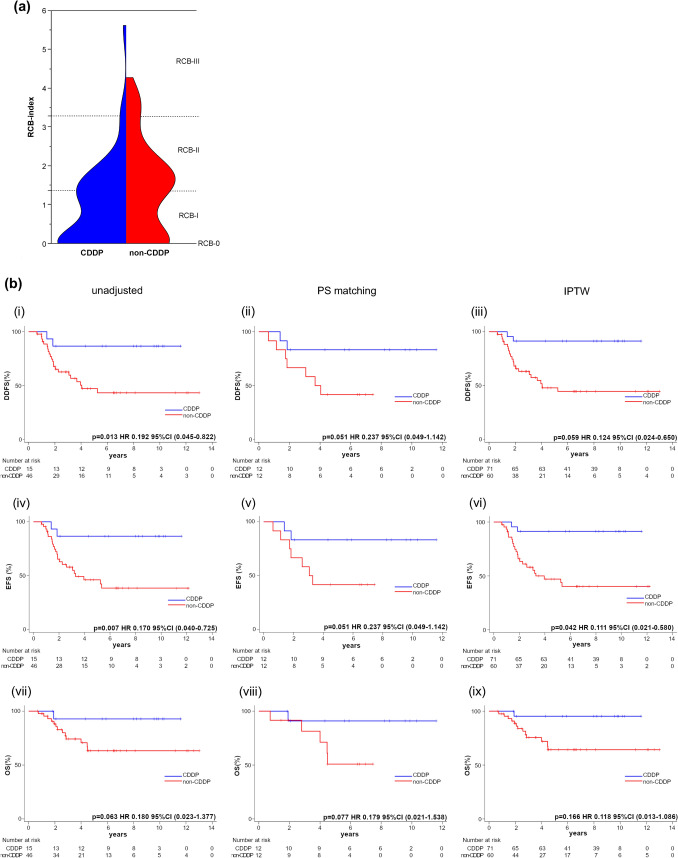
**a** Violin plot showing the percentage distribution of RCB index values for the CDDP and non-CDDP groups. The blue area represents the CDDP group, and the red area represents the non-CDDP group; the areas of both groups are shown as equal. The horizontal axis shows the proportion of patients in each group, with each RCB index value corresponding to the vertical axis. **b** Survival curves of the RCB class II/III group of CDDP and non-CDDP groups analyzed for (i–iii) distant disease-free survival (DDFS), (iv-vi) TNBC-related event-free survival (EFS), and (vii-ix) overall survival. (i, iv, and vii) Are unadjusted, (ii, v, and viii) are adjusted by the propensity score matching method, and (iii, vi, and ix) are adjusted by the inverse probability of the treatment weighting method. Each survival curve was compared using the log-rank method

Figure 3a shows a violin plot for the percentage distribution of RCB index values in the CDDP and non-CDDP groups. The CDDP group had a higher percentage of patients at RCB-0/I, and there were more patients with lower RCB index values within the RCB-II group than in the non-CDDP group. One patient in the CDDP group with cStage IIIc disease had a high RCB index of 5.55. The total population included four patients with cStage IIIc, three of whom were in the CDDP group and two in RCB class 0/I. One patient in the non-CDDP group had an RCB class of II or above. Figure S1 in Online Resource 1 compares the two groups according to RCB class, showing that the percentage of patients at RCB-0 in the CDDP group was significantly higher than in the non-CDDP group. Contrastingly, the percentage of patients at RCB-II in the CDDP group was significantly lower than in the non-CDDP group. The pCR rate was also favorable in the CDDP group (55.8% in the CDDP group and 31.5% in the non-CDDP group).

The survival curves of the RCB class II/III CDDP and non-CDDP groups are shown in Fig. 3b. Table 3 presents the patient characteristics after adjustment for PS matching and IPTW. Regarding DDFS, the log-rank test showed a significantly better prognosis for the CDDP group than the non-CDDP group in the unadjusted analysis; the PS and IPTW analyses showed similar tendencies, although the difference was not statistically significant. The 3-year and 5-year DDFS rates in the unadjusted analysis were 86.7% for the CDDP group and 60.4% and 45.5%, respectively, for the non-CDDP group. As for EFS, the log-rank test showed a significantly better prognosis for the CDDP group than for the non-CDDP group in the unadjusted and IPTW methods, and the PS matching method showed a similar tendency. However, the difference did not reach statistical significance. The 3-year and 5-year EFS rates in the unadjusted group were 86.7% in the CDDP group and 58.0% and 46.2%, respectively, in the non-CDDP group. For OS, the log-rank test showed a tendency for better prognosis in the CDDP group than in the non-CDDP group in the unadjusted, PS matching, and IPTW methods, but there were no significant differences. The 3-year and 5-year OS rates in the unadjusted analysis were 92.9% in the CDDP group and 74.2% and 63.2%, respectively, in the non-CDDP group.

**Table 3:** Patient characteristics with and without adjustment for propensity score (PS) matching and inverse probability of treatment weighting (IPTW) used in the survival sub-analyses for RCB class

Characteristics	Overall			PS matching			IPTW		
	CDDP (*n* = 15)	Non-CDDP (*n* = 46)	Standardized difference	CDDP (*n* = 12)	Non-CDDP (*n* = 12)	Standardized difference	CDDP (*n* = 71)	Non-CDDP (*n* = 60)	Standardized difference
Age									
Mean (SD)	49.6 (9.80)	53.9 (11.85)	0.39	49.3 (10.14)	50.7 (11.77)	0.12	55.1 (24.30)	52.7 (13.76)	0.12
cT									
2–4	9 (60.0%)	37 (80.4%)	0.46	9 (75.0%)	7 (58.3%)	0.36	57 (79.4%)	46 (76.5%)	0.07
1	6 (40.0%)	9 (19.6%)	0.46	3 (25.0%)	5 (41.7%)	0.36	15 (20.6%)	14 (23.5%)	0.07
cN									
Positive	9 (60.0%)	19 (41.3%)	0.38	7 (58.3%)	5 (41.7%)	0.34	23 (32.4%)	27 (44.1%)	0.24
Negative	6 (40.0%)	27 (58.7%)	0.38	5 (41.7%)	7 (58.3%)	0.34	48 (67.6%)	34 (55.9%)	0.24
RCB									
Mean (SD)	2.2 (1.10)	2.3 (0.84)	0.09	2.4 (1.17)	2.3 (0.87)	0.1	2.2 (1.96)	2.3 (0.97)	0.07

### Sensitivity analysis 1: effects of postoperative oral 5-FU administration

The survival curves of the CDDP and non-CDDP groups, excluding the eligible patients taking oral 5-FU (capecitabine, S-1, or UFT) postoperatively, are shown in Fig. S2 of Online Resource 1. Table S3 of Online Resource 1 shows the patient characteristics after each adjustment for this analysis. 15% of patients in the CDDP group and 13% in the non-CDDP group received postoperative oral 5-FU.

Regarding DDFS and EFS, the log-rank test showed a significantly better prognosis for the CDDP group than for the non-CDDP group in the unadjusted, PS matching, and IPTW methods. The 3-year and 5-year DDFS rates in the unadjusted analysis were 97.4% for the CDDP group and 80.5% and 73.6%, respectively, for the non-CDDP group. The 3-year and 5-year EFS rates in the unadjusted analysis were 94.8% for the CDDP group and 80.6% and 70.3%, respectively, for the non-CDDP group. As for OS, the log-rank test showed a similar tendency between the CDDP and non-CDDP groups in the unadjusted, PS matching, and IPTW methods, but the differences were not statistically significant. The 3-year and 5-year OS rates in the unadjusted analysis were 94.7% for the CDDP group and 86.6% and 80.7%, respectively, for the non-CDDP group.

Regarding LMFS, the log-rank test showed a tendency for a better prognosis in the CDDP group than in the non-CDDP group. The HR could not be calculated because the CDDP group had no liver metastatic recurrence.

### Sensitivity analysis 2: redefinition of EFS

The EFS curves of the CDDP and non-CDDP groups for sensitivity analysis 2 are presented in Fig. S3 of Online Resource 1. Events such as second primary breast cancer and cancer of other organs were included; however, the log-rank test still showed a significantly better prognosis for the CDDP group than the non-CDDP group in the unadjusted, PS matching, and IPTW analyses, matching the results using the original EFS definition (Fig. 2d–f). The 3-year and 5-year EFS rates in the unadjusted analysis were 90.6% for the CDDP group and 77.0% and 65.1%, respectively, for the non-CDDP group.

### Clinical response and recurrence pattern in a subset of patients with BRCA 1/2

In this study, nine of 138 patients had mutations in BRCA1/2, two patients were homologous recombination deficiency (HRD) positive (no mutation in BRCA1/2), 28 patients had no mutations in BRCA1/2, and 99 patients were not tested for BRCA1/2 mutation status. The clinical response and recurrence pattern of the 11 patients with a BRCA1/2 mutation or HRD-positive status are summarized in Table S6 of Online Resource 1. Eight of the eleven patients were in the CDDP group and all were distant disease free, although one developed second primary breast cancer 9.77 years after surgery, who carried a BRCA1 mutation. Three of these patients were in the non-CDDP group, two of whom were treated with a CBDCA regimen and were recurrence free. The remaining patient was also in the non-CDDP and no CBDCA and developed lung metastasis 1.67 years after surgery.

### Tumor-infiltrating lymphocytes are associated with prognosis in CDDP-treated patients

Tumor-infiltrating lymphocytes (TILs) may predict preoperative chemotherapy efficacy and prognosis [23, 24]. Therefore, we investigated the prognostic relevance of TILs in 47 patients for whom samples were available in this study. The patient’s background is shown in Table S7 of Online Resource 1.

**Fig. 4:**
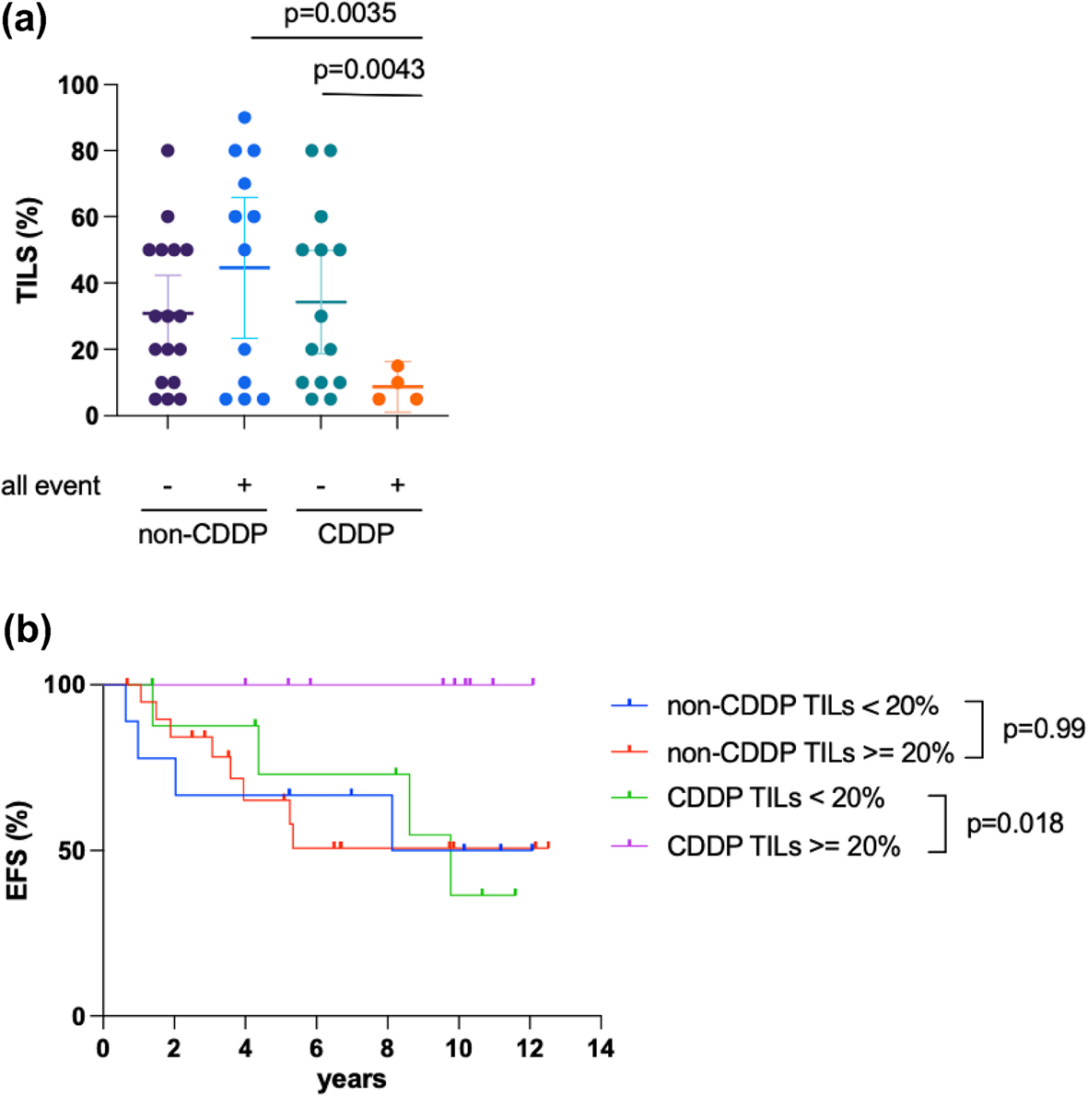
**a** %TILs of the non-CDDP and the CDDP groups by all breast cancer events. TILs were evaluated on HE-stained specimens before preoperative chemotherapy. **b** Survival curves of Non-CDDP and CDDP groups by the degree of TILs for EFS. Each survival curve was compared using the log-rank method

TILs were evaluated in both the non-CDDP and the CDDP groups, segmented by all breast cancer events. In the CDDP group, patients without all breast cancer events demonstrated significantly lower TILs than those with such events (Fig. 4a). Furthermore, we assessed the correlation between EFS and High-TILs versus Low-TILs. Based on previous papers, the cut-off value was set at 20% [25]. No significant difference was observed in the non-CDDP group (Fig. 4b). However, in the CDDP group, a significant improvement in prognosis was noted in the High-TILs group (Fig. 4b *P*=0.018, 95% CI 0.012–0.66, HR 0.09). These findings are consistent with prior TILs reports, suggesting the involvement of immune cells within the tumor immune microenvironment in the efficacy of CDDP treatment [26].

### Elimination rate constant and half-life of platinum in serum

The change in serum platinum concentration over time since the last dose is shown in Figure S4 of Online Resource 1. Since more than one serum sample was available for some patients, a total of 14 serum samples were measured for patients who received CDDP and 15 samples were measured for those who received CBDCA. The detection limit for serum platinum of the system was 0.25 ng/ml. The platinum concentration of the serum of patients in the CDDP group was measurable from 12 to 27 months after the last dose, but the concentration was below the detection sensitivity of mass spectrometer at months 38, 39, and 96 after the last dose. In contrast, the platinum concentration of the serum of patients in the CBDCA group was below the detection sensitivity starting at the 17 months after the last dose, but was measurable from 0 to14 months after the last dose. The elimination constant rates were 0.054 month-1 and 0.385 month-1, and the half-lives of CDDP and CBDCA were determined to be 12.95 and 1.80 months, respectively.

## Discussion

This retrospective observational study revealed a significantly better prognosis in patients who received NAC, including CDDP, than in those who did not in the CDDP group for the primary endpoint of DDFS as well as EFS, and the same tendency was observed for OS in patients with TNBC treated with NAC. This prognostic advantage of CDDP was also observed in the sub-analysis for the RCB-II/III group, which is associated with a poor prognosis [27]. These results may provide novel evidence of the usefulness of CDDP in TNBC treatment.

The KEYNOTE-522 trial used a regimen of paclitaxel and carboplatin plus pembrolizumab, followed by doxorubicin and cyclophosphamide plus pembrolizumab as preoperative treatment, surgery, and then adjuvant pembrolizumab for another nine cycles after surgery. Neoadjuvant pembrolizumab and chemotherapy significantly increased the RCB-0 class rate, and the addition of pembrolizumab postoperatively improved EFS [8]. However, the prognosis of the RCB-II/III group was still not sufficiently favorable while the introduction of pembrolizumab [28]. Our results showed that the 3-year EFS rate of the RCB-II/III group receiving CDDP was 86.7%, suggesting a possibility of further improvement in survival when pembrolizumab and CDDP are combined; promising candidate for chemotherapy in combination with pembrolizumab. Additionally, the choice of preoperative chemotherapy for cStage I TNBC remains currently controversial; however, our results showed no cStage I distant metastatic recurrences in the CDDP group, which occurred in 19.0% of cStage I patients in the non-CDDP group (Table S2 of Online Resource 1). These results suggest that preoperative chemotherapy, including CDDP, may benefit cStage I TNBC patients not indicated for pembrolizumab treatment.

In addition to anticancer effects and inhibition of DNA cross-linking and mitosis, leading to the apoptosis of cancer cells, CDDP also exhibits immunomodulatory effects, including increased MHC class I expression, recruitment, and proliferation of effectors, the increased lytic activity of cytotoxic effectors, and downregulation of the immunosuppressive microenvironment [29]. CBDCA is also a platinum agent but has a different structural formula, which suggests that CDDP binds irreversibly to plasma albumin and may interact irreversibly with tissue proteins and DNA. Contrastingly, CBDCA binds reversibly to plasma proteins [30]. The difference between these two platinum agents in antitumor immunity remains still unclear; however, the TONIC trial of metastatic TNBC revealed that both CDDP and doxorubicin significantly enhanced the response to anti-PD-L1 therapy with nivolumab [31]. Therefore, CDDP promotes antitumor immune activity.

Platinum agents are effective in patients with TNBC, especially in those with BRCA1 mutations and homologous recombination deficiency [32, 33]. However, there are few studies on regimens that include CDDP, especially regimens combined with anthracyclines and taxanes in the preoperative setting. Although the number of patients was small and follow-up periods for some patients were short, we demonstrated that patients with BRCA1/2 mutation in the CDDP group maintained a distant disease-free status.

Since the CREATE-X study clearly showed that patients receiving postoperative oral 5-FU have better outcomes [19], we decided to perform a sensitivity analysis 1 to remove the effect of 5-FU from the analysis. Oral 5-FU administered to patients at high risk of recurrence (15% in the CDDP group and 13% in the non-CDDP group) resulted in fewer events. However, there were still significant differences in DDFS and EFS and no change in the tendency for OS and LMFS.

In sensitivity analysis 2, the definition of events included the recurrence of primary TNBC and the development of 2nd primary BC and cancer in other organs. However, the tendency of EFS analysis remained unchanged, supporting the preliminary analysis.

This study detected no liver metastatic events in the CDDP group. Liver metastases are often lethal, and the predicted 5-year survival rate of breast cancer patients is 8.5% [34]. Fewer liver metastases may be associated with a better prognosis in the CDDP group. The mechanism by which CDDP reduces liver metastases is unclear; however, the CDDP group had significantly fewer liver metastases and significantly better LMFS than the non-CDDP group. Furthermore, previous studies have shown that liver metastases from breast cancer respond less well to immune checkpoint therapy than metastases from other sites [35]. Notably, several mechanisms have been demonstrated to explain immune tolerance in the liver, including expression of PD-L1 by liver sinusoidal endothelial cells to induce and maintain T-cell tolerance and activation of regulatory T cells by Kupffer cells [36]. A hypothesis that CDDP has the potential to inhibit liver metastases is warranted to investigate further and a clinical study using the combination with immune checkpoint therapy and CDDP is needed.

We also found that TILs served as a prognostic marker for CDDP treatment for preoperative chemotherapy. The correlation of TILs as a prognostic factor in TNBC aligns with existing literature. Similarly, several previous studies have indicated the prognostic and predictive significance of TILs in breast cancer, including TNBC [23, 37]. Particularly, high levels of TILs are associated with an increased likelihood of response to neoadjuvant chemotherapy and better overall survival in patients with TNBC [38]. Our findings further substantiate the role of TILs as an indicator of response to platinum-based therapy in TNBC. Platinum-based agents, including cisplatin, exert their anticancer effects by inducing DNA damage, which triggers the immune response [39]. TILs may reflect a pre-existing antitumor immune response, which could be amplified by platinum-based chemotherapy, leading to improved outcomes.

Our study highlights the prognostic value of TILs in TNBC patients receiving preoperative platinum-based therapy. It adds to the growing body of evidence supporting the role of the immune microenvironment in cancer progression and treatment response.

Furthermore, although there have been several reports on the long-term effects of serum platinum after CDDP treatment for germ-line cell tumors [40, 41], knowledge of long-term platinum retention in patients treated with CBDCA is very limited. In order to determine the differential impact of CDDP and CBDCA on long-term prognosis, we measured serum platinum concentrations over time in patients treated with CDDP and CBDCA using a mass spectrometer and calculated the elimination constant and half-life, regardless of the subtype of breast cancer. The half-life of platinum in the serum of patients in the CBDCA group was 1.8 months, while that of patients in the CDDP group was approximately 13 months, indicating that platinum in serum remained longer in the CDDP group. Although this analysis was limited by a small number of patients in each group, varying periods of serum collection, and the inclusion of breast cancer patients other than TNBC, the results suggest that the long-term persistence of serum platinum in CDDP-treated patients may have contributed to the more favorable prognosis of the CDDP group.

This study had some limitations. First, this was a retrospective study, and selection bias regarding the choice of patients using CDDP at Kyoto University Hospital cannot be ruled out. However, there was no selection bias since all patients in the other centers were included in the non-CDDP group. Since approximately 60% of the non-CDDP group consisted of patients outside Kyoto University Hospital, the effect of selection bias on the results of this study is limited. The second limitation is the difference in age between the CDDP and non-CDDP groups. Age is one of the factors that significantly affects the efficacy of chemotherapy. Therefore, this difference in patient background cannot be ignored. In the stratified analysis of this study, there was a similar trend toward a difference in DDFS between the CDDP and non-CDDP groups in patients aged 50 years or older (Fig. S5). Third, this was a retrospective study and included a variety of regimens in three medical centers for both the CDDP and non-CDDP groups. Nevertheless, there was no difference in survival outcomes of the non-CDDP group between hospitals (Fig. S6). Additionally, there was no significant difference in the prognosis and distribution of the RCB class and DDFS in the non-CDDP group between Kyoto University Hospital and the other two medical centers (data not shown). Furthermore, no difference was observed in the proportion of patients who received anthracycline- or taxane-based regimens between the CDDP and non-CDDP groups. Therefore, we did not consider the variation in regimens to impact the study results significantly. From this study’s point of feasibility profile, no significant difference in treatment completion rates was detected between the CDDP and non-CDDP groups (Table 1). Based on the incidence of severe side effects and treatment interruptions with the CDDP regimen (Table S3 of Online Resource 1), we considered regimens with CDDP as safe as those without CDDP. As CDDP is a long-standing anticancer drug with a well-known side effect profile, adequate antiemetics and infusions have been administered to reduce side effects [42, 43]. Fourth, since this study was conducted in a retrospective setting, the number of cases was not designed to show a statistically significant difference, and the sample size may be small. Therefore, we used PS matching and IPTW to analyze survival curves to compensate for the small sample size. Fifth, since CBDCA was not covered by insurance for outpatient chemotherapy treatment of TNBC in Japan at the time of this study, only three patients in the non-CDDP group were treated with CBDCA. Therefore, we were not able to perform a comparison between the CDDP and CBDCA groups. Furthermore, the pCR rate in the CDDP group was 55.8%, whereas it was only 31.4% in the non-CDDP group. The pCR rate in the non-CDDP group of this study was lower than those reported (53–60%) in previous randomized control studies using CBDCA-containing regimens [9, 44, 45]. This difference may be due to the low proportion of patients who received CBDCA-based treatment regimen in the non-CDDP group in our study, which was 3.5% (3/86).

Collectively, our study showed that neoadjuvant CDDP-containing regimens improve the prognosis of patients with operable TNBC. Furthermore, the subgroup analysis indicated that the CDDP-containing regimen could improve prognosis in patients with residual disease after NAC and liver metastasis.

CDDP is a promising drug that has attracted attention owing to its interactions with immune checkpoint inhibitors and may play an essential role in future treatment strategies for operable TNBC. Based on the results of this study, a prospective study is warranted to elucidate the role of CDDP in the treatment of TNBC, which could lead to improved survival.

### Supplementary Information

Below is the link to the electronic supplementary material.

Supplementary file1 (DOCX 746 kb)

## Data Availability

The datasets generated during and/or analyzed during the current study are not publicly available due to their containing information that could compromise the privacy of research participants but are available from the corresponding author upon reasonable request.

## Abbreviations

TNBC: Triple-negative breast cancer
RD: Residual disease
NAC: Neoadjuvant chemotherapy
OS: Overall survival
CDDP: Cisplatin
DDFS: Distant disease-free survival
PSM: Propensity score matching
IPTW: Inverse probability of treatment weighting
RCB: Residual cancer burden
CBDCA: Carboplatin
EFS: Event-free survival
JRC: Japanese Red Cross
LMFS: Liver metastasis-free survival
PS: Propensity score
