# Supplementary material for: Comparison of cisplatin-based versus standard preoperative chemotherapy in patients with operable triple-negative breast cancer: propensity score matching and inverse probability of treatment weighting analysis
Source: Breast Cancer Res Treat. 2023 Dec 21;204(2):261–75. doi: 10.1007/s10549-023-07163-z (PMC10948496; doi:10.1007/s10549-023-07163-z)
Supplement: Supplementary file 1 — Supplementary file1 (DOCX 746 kb) [file 10549_2023_7163_MOESM1_ESM.docx]

**Online Resource 1**

Supplementary Material for

*Breast Cancer Research and Treatment*

**Cisplatin-based preoperative chemotherapy reduces residual tumor volume and improves prognosis in patients with triple-negative breast cancer**

Ayane Yamaguchi, Kosuke Kawaguchi^*^, Yurina Maeshima, Akiyoshi Nakakura, Tatsuki R. Kataoka, Shunsaku Nakagawa, Atsushi Yonezawa, Sachiko Takahara, Masahiro Takada, Masahiro Kawashima, Nobuko Sakita-Kawaguchi, Takeshi Kotake, Eiji Suzuki, Hanako Shimizu, Masae Torii, Satoshi Morita, Hiroshi Ishiguro, Masakazu Toi

*Corresponding author

Kosuke Kawaguchi M.D., Ph.D.

Kyoto University Graduate School of Medicine, 54 Shogoin Kawaramachi, Sakyo-ku, Kyoto 606-8507, Japan

E-mail: kkosuke@kuhp.kyoto-u.ac.jp

TEL: +81-75-751-3660; FAX: +81-75-751-3616

(a)


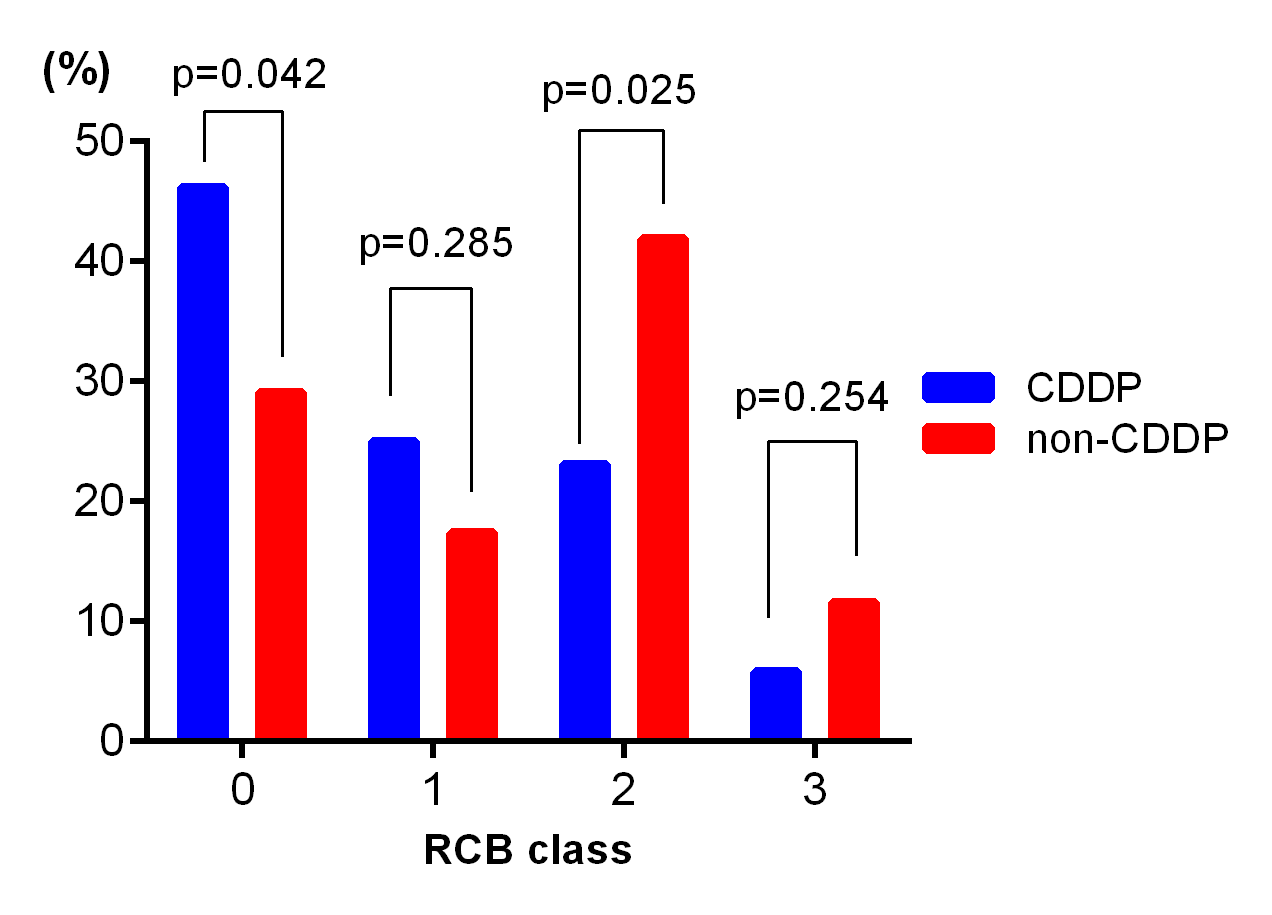


(b)

**Fig. S1**

(a) Percentage of patients in each RCB class in the CDDP and non-CDDP groups compared using two-tailed *t*-tests. (b) Percentage of patients in pCR rate in the CDDP and non-CDDP groups.


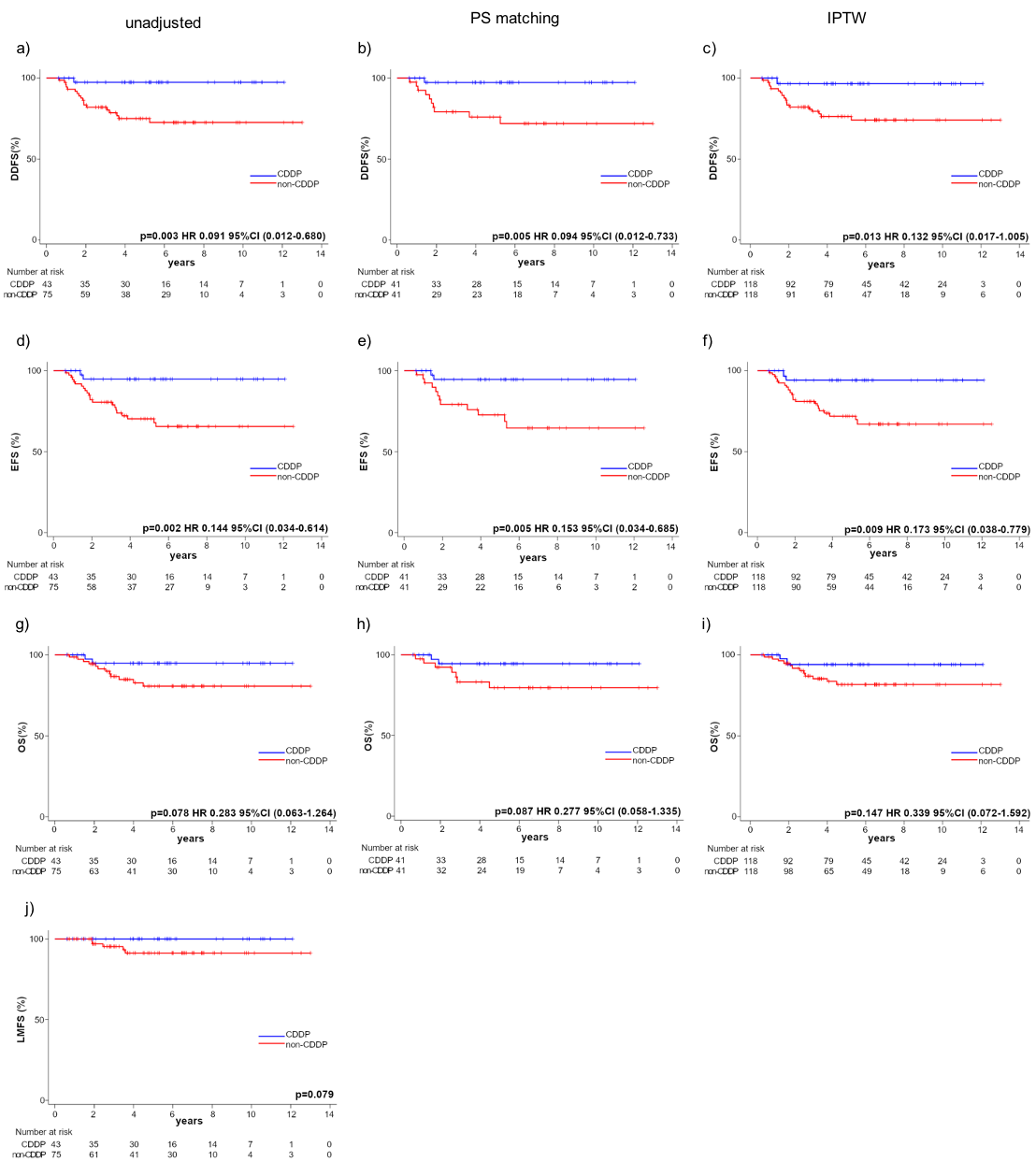
 **Fig. S2** Survival curves in sensitivity analysis of the CDDP and non-CDDP groups, excluding patients who received postoperative oral 5-FU, for (a–c) distant disease-free survival (DDFS), (d–f) event-free survival (EFS), (g–i) overall survival (OS), and (j) liver metastasis-free survival (LMFS). (a, d, g, and j) are unadjusted; (b, e, and h) are adjusted using the propensity score matching method; and (c, f, and i) are adjusted using the inverse probability of treatment weighting method. Each survival curve was compared using the log-rank method


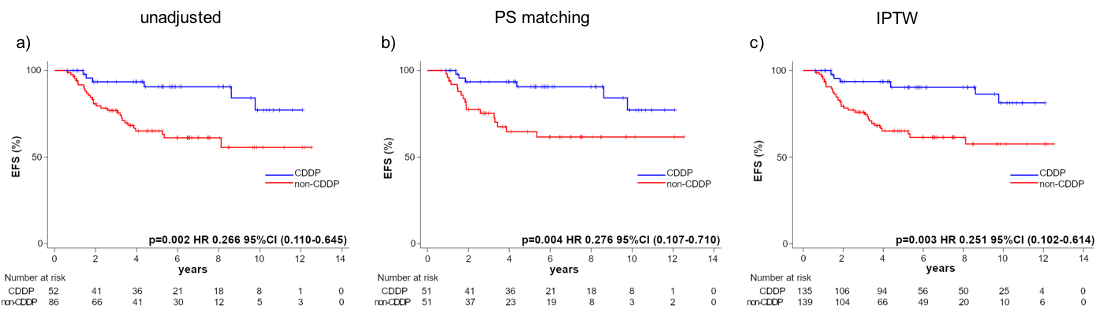
**Fig. S3** Survival curves in sensitivity analysis for event-free survival (EFS) of the CDDP and non-CDDP groups for all patients. This analysis includes second primary breast cancer and cancer of other organs not associated with the original TNBC as events. (a) are unadjusted, (b) are adjusted using the propensity score matching method, and (c) are adjusted using the inverse probability of treatment weighting method. Each survival curve was compared using the log-rank method.


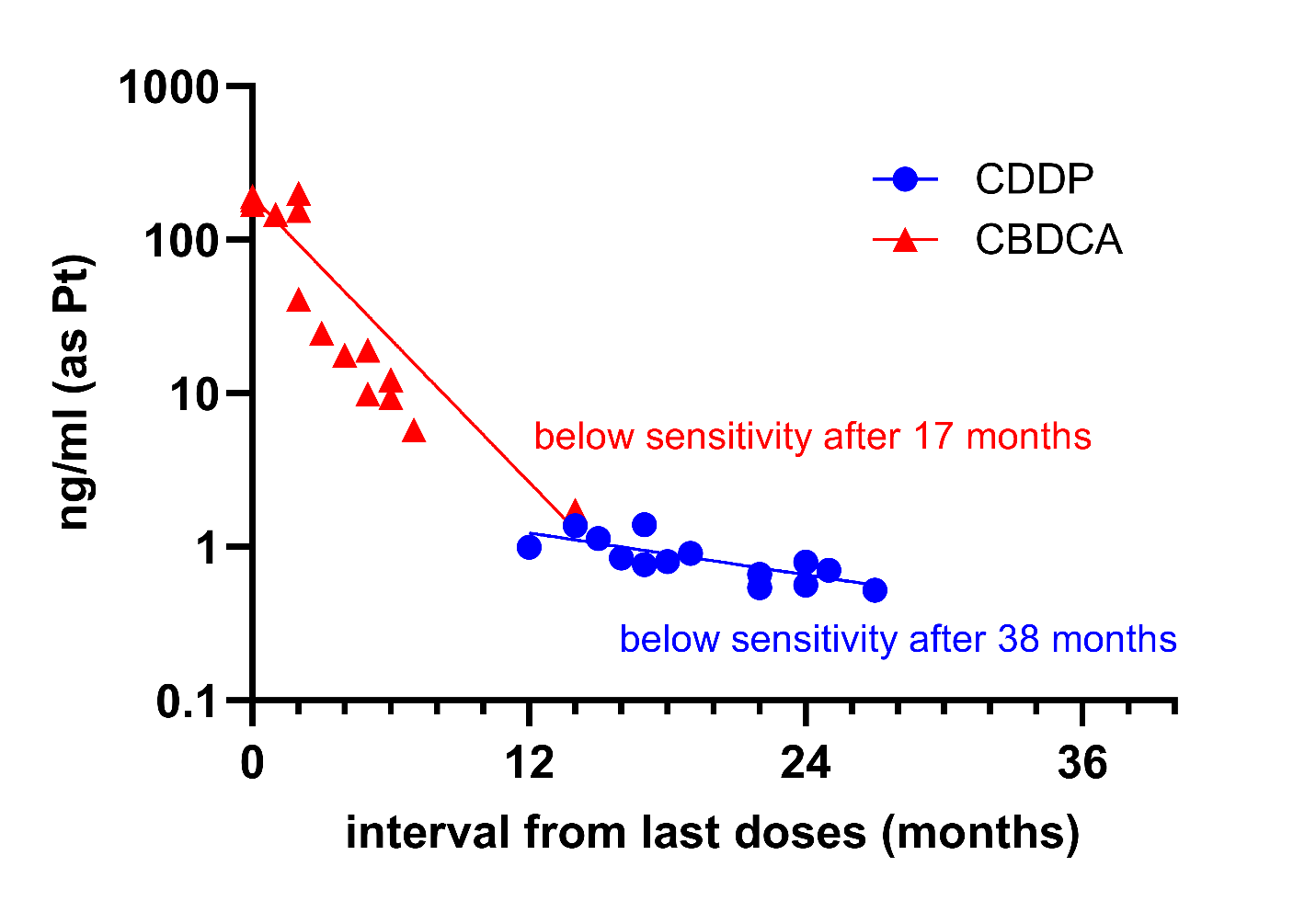


**Fig. S4** A graph plotting serum Pt concentration by time since the last dose. The detectable concentration of Pt in serum was 0.4 ng/ml. The Pt concentration of the serum of patients in the CDDP group were measurable from 12-27 months after the last dose, but it was below the detectable sensitivity at months 38, 39, and 96 after the last dose. In contrast, the Pt concentration of the serum of patients in the carboplatin CBDCA group had below the detectable sensitivity after the 17 months after the last dose, and those were measurable from 0-14 months after the last dose.


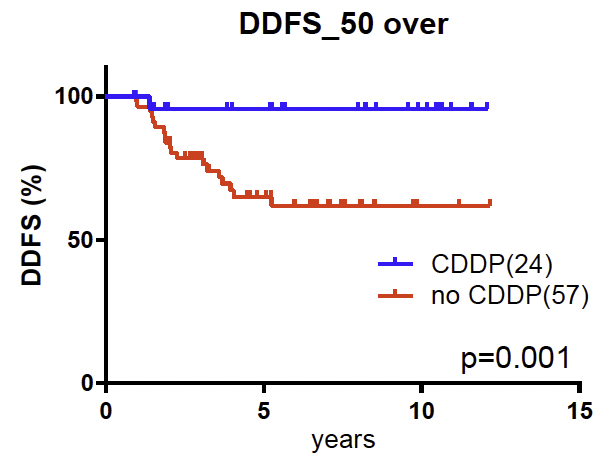


**Fig. S5** Survival curves in sensitivity for distant disease-free survival (DDFS) of the CDDP and non-CDDP groups for groups over 50 years old.

**
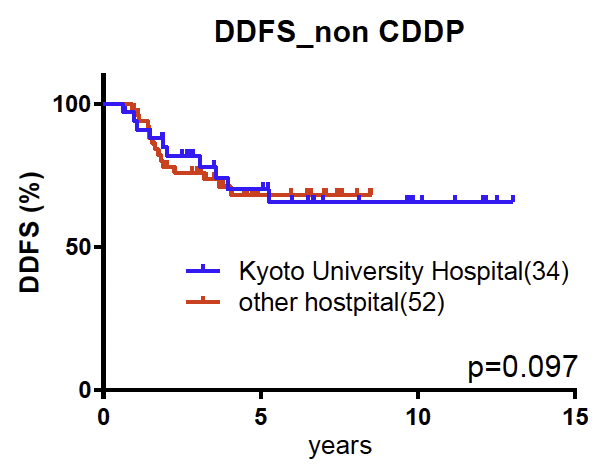
**

**Fig. S6** Survival curves in sensitivity for distant disease-free survival (DDFS) of the Kyoto-University Hospital and other hospital for non-CDDP groups. Each survival curve was compared using the log-rank method

**Table S1**

Chemical structures and pharmacokinetics of CDDP and CBDCA following intravenous infusion.

|  | **CDDP** | **CBDCA** |
| --- | --- | --- |
| chemical structures | 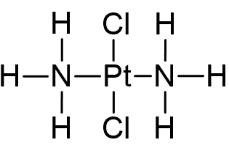 | 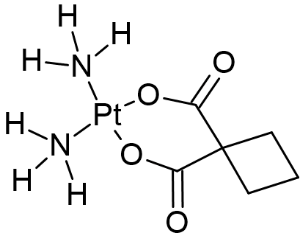 |
| Total Platinum |  |  |
| t_1/2α_(min) | 14-49 | 12-98 |
| t_1/2β_(h) | 0.7-0.8 | 1.7-5.9 |
| t_1/2γ_(h) | 24-127 | 8.2-40 |
| **Protain Binding (%)** | >90 | 24-50 |
| **Urinary Excretion (%)** | 23-50 | 54-82 |
| t_1/2α_, t_1/2β_, and t_1/2γ_ = early, mid, and late half-lives. | | |

**Table S2**

List of patients who were measured serum platinum concentrations after receiving a regimen containing CDDP or CBDCA

| **Age** | **CDDP/CBDCA** | **subtype** | **regimen** | **months from last dose** |
| --- | --- | --- | --- | --- |
| 46 | CDDP | TNBC | TPx4 | 27, 96 |
| 54 | CDDP | TNBC | TPx4 | 22 |
| 54 | CDDP | TNBC | TPx4 | 24 |
| 32 | CDDP | TNBC | TPx4 | 22 |
| 43 | CDDP | TNBC | TPx4 | 24 |
| 57 | CDDP | TNBC | TPx4 | 17 |
| 54 | CDDP | TNBC | TPx4 | 17 |
| 56 | CDDP | TNBC | TPx4 | 12 |
| 50 | CDDP | TNBC | TPx4 | 16 |
| 60 | CDDP | TNBC | TPx4 | 18 |
| 29 | CDDP | TNBC | TPx4 | 14 |
| 34 | CDDP | TNBC | TPx4 | 15 |
| 49 | CDDP | TNBC | TPx4 | 39 |
| 46 | CDDP | TNBC | TPx4 | 25, 38 |
| 58 | CDDP | TNBC | GEMCDDPx4 | 19 |
| 35 | CBDCA | HER2-positive | PacCbH (Cbx4) | 0, 19 |
| 61 | CBDCA | HER2-positive | PacCbH (Cbx5) | 1, 14 |
| 70 | CBDCA | HER2-positive | PacCbH (Cbx5) | 0, 5, 7 |
| 49 | CBDCA | HER2-positive | TCbH (Cbx6) | 2, 17 |
| 44 | CBDCA | HER2-positive | TCbH (Cbx6) | 0, 5 |
| 33 | CBDCA | HER2-positive | TCbH (Cbx6) | 6, 18 |
| 41 | CBDCA | HER2-positive | TCbH (Cbx6) | 4 |
| 47 | CBDCA | HER2-positive | TCbH (Cbx6) | 2, 6 |
| 62 | CBDCA | HER2-positive | TCbHP (Cbx4) | 3, 20 |
| 42 | CBDCA | TNBC | GEMCBDCA (Cbx6) | 2 |
| T=docetaxel P=CDDP GEM=gemcitabine Pac=paclitaxel Cb=carboplatin H=harceptin HP=harceptin+pertuzumab | | | | |

**Table S3**

Toxicity profiles of the CDDP groups and non-CDDP group from Kyoto University Hospital above Grade 3 and toxicity related to dose intensity.

|  | CDDP group　　　　　　 　(52 patients, 403 cycles) | |  | non-CDDP group (34 patients, 402 cycles) | |
| --- | --- | --- | --- | --- | --- |
|  | n | % |  | n | % |
| **Grade3/4 adverse event (all cycles)** | 32 | 7.9% |  | 53 | 13.2% |
| **Haematologic adverse events** |  |  |  |  |  |
| anemia | 8 | 2.0% |  | 2 | 0.5% |
| neutropenia | 9 | 2.2% |  | 33 | 8.2% |
| febrile neutropenia | 9 | 2.2% |  | 7 | 1.7% |
| lymphocyte decreased | 0 | 0.0% |  | 2 | 0.5% |
| thrombocytopenia | 1 | 0.2% |  | 1 | 0.2% |
| **Non-haematologic adverse events** |  |  |  |  |  |
| elevated AST/ALT | 2 | 0.5% |  | 11 | 2.7% |
| nausea/vomitting | 3 | 0.7% |  | 21 | 5.2% |
| rash | 0 | 0.0% |  | 1 | 0.2% |
| peripheral neuropathy | 0 | 0.0% |  | 1 | 0.2% |
| palmar-plantar erythrodysesthesia syndrome | 0 | 0.0% |  | 3 | 0.7% |
| diarrhea | 0 | 0.0% |  | 1 | 0.2% |
| dehydration | 0 | 0.0% |  | 1 | 0.2% |
| hypokalemia | 0 | 0.0% |  | 1 | 0.2% |
| **Adverse event leading to death** | 0 | 0.0% |  | 0 | 0.0% |
| **Adverse event leading to discontinuation** | 11 | 2.7% |  | 4 | 1.0% |
| **Adverse event leading to dose reduction** | 5 | 1.2% |  | 28 | 7.0% |

**Table S4** Characteristics of patients with distant metastatic recurrence

| **Age** | **Use of CDDP** | **Clinical stage** | **cT** | **cN** | **RCB class** |
| --- | --- | --- | --- | --- | --- |
| 53 | non-CDDP | I | 1c | 0 | 2 |
| 32 | non-CDDP | I | 1c | 0 | 3 |
| 70 | non-CDDP | I | 1 | 0 | 2 |
| 54 | non-CDDP | I | 1 | 0 | 2 |
| 61 | non-CDDP | IIA | 2 | 0 | 2 |
| 50 | non-CDDP | IIA | 2 | 0 | 2 |
| 50 | non-CDDP | IIA | 2 | 0 | 2 |
| 51 | non-CDDP | IIA | 2 | 0 | 2 |
| 58 | non-CDDP | IIA | 2 | 0 | 2 |
| 69 | non-CDDP | IIA | 1 | 1 | 3 |
| 61 | non-CDDP | IIB | 2 | 1 | 2 |
| 61 | non-CDDP | IIB | 2 | 1 | 2 |
| 40 | non-CDDP | IIB | 2 | 1 | 3 |
| 57 | CDDP | IIIA | 3 | 1 | 2 |
| 57 | non-CDDP | IIIA | 3 | 1 | 3 |
| 42 | non-CDDP | IIIA | 3 | 1 | 3 |
| 68 | non-CDDP | IIIA | 3 | 1 | 2 |
| 42 | non-CDDP | IIIA | 3 | 2 | 3 |
| 46 | non-CDDP | IIIA | 1 | 3 | 0 |
| 65 | non-CDDP | IIIB | 4b | 1 | 2 |
| 67 | non-CDDP | IIIB | 4b | 0 | 1 |
| 49 | non-CDDP | IIIB | 4b | 1 | 2 |
| 66 | non-CDDP | IIIB | 4 | 1 | 2 |
| 62 | non-CDDP | IIIB | 4 | 1 | 2 |
| 54 | non-CDDP | IIIB | 4b | 0 | 2 |
| 48 | CDDP | IIIC | 1c | 3 | 3 |
| 65 | non-CDDP | IIIC | 3 | 3 | 2 |

**Table S5** Patient characteristics with and without adjustment for propensity-score (PS) matching and inverse probability of treatment weighting (IPTW) used in the sensitivity analysis for postoperative oral 5-FU administration

| Characteristic | | Overall | | | PS matching | | | IPTW | | |
| --- | --- | --- | --- | --- | --- | --- | --- | --- | --- | --- |
|  |  | CDDP  (n =43) | non-CDDP (n = 75) | Standardized difference | CDDP  (n = 41) | non-CDDP (n = 41) | Standardized difference | CDDP  (n = 118) | non-CDDP (n = 118) | Standardized difference |
| Age | Mean (SD) | 47.6 (11.35) | 54.4 (11.47) | 0.60 | 48.4 (11.03) | 49.1 (10.74) | 0.06 | 51.9 (19.53) | 52.0 (14.89) | 0.01 |
| cT | 2–4 | 31 (72.1%) | 53 (70.7%) | 0.03 | 29 (70.7%) | 29 (70.7%) | 0.00 | 87 (73.5%) | 84 (71.1%) | 0.05 |
|  | 1 | 12 (27.9%) | 22 (29.3%) | 0.03 | 12 (29.3%) | 12 (29.3%) | 0.00 | 31 (26.5%) | 34 (28.9%) | 0.05 |
| cN | positive | 13 (30.2%) | 25 (33.3%) | 0.07 | 13 (31.7%) | 12 (29.3%) | 0.05 | 42 (35.9%) | 39 (33.0%) | 0.06 |
|  | negative | 30 (69.8%) | 50 (66.7%) | 0.07 | 28 (68.3%) | 29 (70.7%) | 0.05 | 76 (64.1%) | 79 (67.0%) | 0.06 |

**Table S6** Clinical response and recurrence pattern in a subset of patients with BRCA 1/2 mutations

| Age | mutated gene | CDDP/CBDCA | Clinical stage | cT | cN | RCB class | Recurrence | follow-up period (years) |
| --- | --- | --- | --- | --- | --- | --- | --- | --- |
| 31 | BRCA1 | CDDP | IIA | 2 | 0 | 2 | no recurrence | 4.32 |
| 39 | BRCA1 | CDDP | I | 1c | 0 | 2 | 2nd primary breast cancer (DFI: 9.77years) | 9.77 |
| 46 | BRCA1 | CDDP | IIA | 2 | 0 | 0 | no recurrence | 2.58 |
| 47 | BRCA1 | CBDCA | I | 1c | 0 | 2 | no recurrence | 3.53 |
| 54 | BRCA1 | CDDP | IIA | 2 | 0 | 0 | no recurrence | 5.26 |
| 56 | BRCA1 | CDDP | IIA | 2 | 0 | 2 | no recurrence | 5.60 |
| 32 | BRCA2 | CDDP | IIA | 2 | 0 | 1 | no recurrence | 6.11 |
| 40 | BRCA2 | non-CDDP/CBDCA | IIB | 2 | 1 | 3 | lung metastasis (DFI: 1.65years) | 1.82 |
| 46 | BRCA2 | CDDP | IIB | 3 | 0 | 0 | no recurrence | 8.55 |
| 33 | HRD positive | CDDP | I | 1c | 0 | 0 | no recurrence | 1.38 |
| 35 | HRD positive | CBDCA | IIA | 2 | 0 | 2 | no recurrence | 0.67 |
|  |  |  |  |  |  |  | Homologous Recombination Deficiency : HRD DFS: Disease Free Interval | |

**Table S7** Patient background that provided an available sample for TIL assessment.

| Characteristics |  | CDDP (n=18) | | Non-CDDP (n=29) | | P value |
| --- | --- | --- | --- | --- | --- | --- |
|  |  | n | % | n | % |  |
| Age | ≤50 | 8 | 44.4 | 10 | 34.5 | *0.548* |
|  | >50 | 10 | 55.6 | 19 | 65.6 |  |
|  | range (median) | 39–68 (51) | | 31–76 (57) | |  |
| cT stage | 1c | 7 | 38.9 | 4 | 13.8 | *0.079* |
|  | 2 | 7 | 38.9 | 17 | 58.6 |  |
|  | 3 | 4 | 22.2 | 4 | 13.8 |  |
|  | 4b–d | 0 | 0.0 | 4 | 13.8 |  |
| cN | negative | 10 | 55.6 | 19 | 65.6 | *0.548* |
|  | positive | 8 | 38.9 | 10 | 31.0 |  |
|  | cN1 | 7 | 0.0 | 9 | 0.0 |  |
|  | cN3a-b | 1 | 5.6 | 1 | 3.4 |  |
| cStage | I | 4 | 22.2 | 3 | 10.3 | *0.459* |
|  | IIA–B | 9 | 50.0 | 19 | 65.6 |  |
|  | IIIA–C | 5 | 27.8 | 7 | 24.1 |  |
| ER status | 0 | 4 | 22.2 | 7 | 75.9 | *1.000* |
|  | 1–9% | 14 | 77.8 | 22 | 24.1 |  |
| *BRCA/*HRD status | *BRCA1/2 mutation* (+)  or HRD positive | 2 | 11.1 | 2 | 6.9 | *0.766* |
|  | no mutation  and HRD negative | 6 | 33.3 | 8 | 27.6 |  |
|  | unknown | 10 | 55.6 | 19 | 65.6 |  |
| Chemotherapy regimen | Anthracycline-based | 14 | 77.8 | 24 | 82.8 | *0.716* |
|  | Taxane-based | 17 | 94.4 | 22 | 75.9 | *0.130* |
| Complete chemotherapy | completed | 16 | 88.9 | 24 | 82.8 | *0.692* |
| Radiotherapy | Yes | 14 | 77.8 | 24 | 82.8 | *0.716* |
|  | None | 4 | 22.2 | 5 | 17.2 |  |
| Adjuvant oral 5-FU | Yes | 3 | 16.7 | 4 | 13.8 | *1.000* |
|  | No/unknown | 15 | 83.3 | 25 | 86.2 |  |
